# Transcriptome Analysis of Environmental Adaptation of Largemouth Bass (*Micropterus salmonides*)

**DOI:** 10.3390/genes16030267

**Published:** 2025-02-24

**Authors:** Yuao Wang, Huan Li, Chuan Li, Weibin Tang, Yanchao Wang, Hongxia Hou

**Affiliations:** 1 College of Chemical Engineering and Biotechnology, Xingtai University, Xingtai 054000, China; 2 Shahe Bureau of Agriculture and Rural Affairs, Xingtai 054100, China; 3 Hebei Key Laboratory of Digital Freshwater Aquaculture Technology, Xingtai University, Xingtai 054000, China

**Keywords:** largemouth bass, transcriptome, differentially expressed genes, stress response

## Abstract

Background/Objectives: The largemouth bass (*Micropterus salmonides*) is a farmed fish of significant economic value, and studying its adaptability is crucial for enhancing the economic benefits of aquaculture. The largemouth bass changes gene expression pattern to rapidly adapt to environmental changes and maintain normal physiological function. Methods: In this study, largemouth bass from two distinct environmental backgrounds—Huzhou and Xingtai—were used as experimental materials, and they have significantly different breeding conditions. Comparative transcriptomics was used to analyze the gene expression patterns in largemouth bass from both backgrounds. Results: In the female, there were 1678 differentially expressed genes, of which 541 were upregulated and 1137 were downregulated. Meanwhile, in the male, there were 1287 differentially expressed genes, including 542 upregulated genes and 745 downregulated genes. The differentially expressed genes were mainly enriched in biological processes such as metabolic process, biological regulation, response to stimulus, developmental process, signaling, reproduction and immune system process. The enriched pathways included carbon metabolism, glycolysis/gluconeogenesis, purine metabolism, biosynthesis of amino acids, starch and sucrose metabolism, fructose and mannose metabolism, pyrimidine metabolism, MAPK signaling pathway, spliceosome, protein processing in the endoplasmic reticulum, ribosome biogenesis in eukaryotes, etc. Conclusions: We speculated that largemouth bass in Xingtai may adapt to the environment by downregulating metabolism- and reproduction-related genes and altering the expression of immune-related genes. Our study provided molecular evidence for the adaptation research of largemouth bass and provided a scientific basis for optimizing largemouth bass breeding technology.

## 1. Introduction

The largemouth bass (LMB; *Micropterus salmoides*; order Perciformes, suborder Percoidei, family Centrarchidae) is a freshwater fish with important economic value [1]. The largemouth bass is favored by consumers and farmers for its delicious meat, fast growth rate and strong disease resistance [2]. Intensive farming is common, which brings great challenges to the sustainable development of aquaculture [3]. The largemouth bass has strong adaptability and shows good survival ability under different breeding conditions. Previous study had shown that adjusting the feeding pattern of largemouth bass can benefit growth and adaptability. Extruded and pelleted diets induced differences in growth performance and nutrient retention of largemouth bass [4]. It has been proposed that, when implementing a scientific strategy for food restriction in largemouth bass, the intrinsic apoptosis pathway, lysosome pathway and endoplasmic reticulum stress pathway might inhibit hepatocyte apoptosis [5]. Feed additives can also enhance the growth performance and adaptability. The by-products of Chinese yam (*Dioscorea polystachya* Turczaninow) used as a feed additive can protect liver and intestine health, increase beneficial bacteria abundance and decrease potential pathogens in largemouth bass [6]. Ursolic acid can significantly improve the growth performance and antiviral and antioxidant capacity of largemouth bass by affecting the abundance of intestinal flora and improve intestinal barrier function [7,8]. Hydrolyzable tannin improved growth performance of largemouth bass and enhanced the hepatic antioxidant capacity and glycolipid metabolism [9]. β-glucan is widely used in aquaculture due to its immunostimulatory effects, which enhanced immunity and regulated the gut microbiota [10]. The combination of β-glucan and Astragalus polysaccharide can effectively resist *Nocardia seriolae* infection of largemouth bass [11]. However, at the molecular level, the adaptability of largemouth bass is mainly reflected in a rapid response to environmental changes. Gene expression patterns change dynamically in response to environmental factors, such as temperature, salinity and oxygen. Therefore, transcriptome analysis is one of the important measures to study the adaptive mechanism.

When exposed to chronic heat, oxidative stress occurred in the head kidney and spleen tissues of largemouth bass, resulting in decreased apoptosis and immune gene expression, decreased blood immune indexes and increased susceptibility to *Aeromonas hydrophila* [12]. Transcriptome sequencing analysis revealed that, after 24 h of survival at 34 °C, there was a significant increase in alternative splicing events and the number of alternative splicing genes [13]. The functions of these genes were primarily enriched in immune-related pathways, including necroptosis, apoptosis and the C-type lectin receptor signaling pathway. Cold exposure significantly downregulated the expression of genes crucial for gonadal development and diminished the vascular network on the ovarian membrane. Cold temperatures have been shown to delay ovarian development in largemouth bass, primarily through their effects on sex hormone synthesis, the process of angiogenesis and the deposition of lipids [14]. The adaptation of largemouth bass to salt stress was achieved by upregulating UDP-glucuronosyltransferase genes (*UGTs*) and glutathione S-transferase genes (*GSTs*) in the kidneys to eliminate toxic substances and mitigate oxidative damage, and the upregulation of genes such as Cystatin A1 favors recovery from kidney injury [15]. Crowding stress may negatively affect fish growth and intestinal integrity by inducing apoptosis and autophagy associated with endoplasmic reticulum stress-mediated unfolded protein response [16]. Dissolved oxygen is an important parameter in aquaculture. Hypoxic stress affects the antioxidant capacity of largemouth bass, resulting in intestinal tissue damage, angiogenesis and structural changes in microflora [17,18]. Moreover, hypoxic stress also altered the morphology and function of mitochondria and the endoplasmic reticulum [19]. The interaction of heat and hypoxia can aggravate oxidative stress, inhibit immunity and promote apoptosis [20]. The largemouth bass can significantly enhance its tolerance to hypoxia by adjusting metabolic strategies and related signal pathways to adapt to different dissolved oxygen environments [21,22,23]. However, in acute hypoxia conditions, patterns of glucose and lipid utilization were altered to provide energy and maintain physiological function [24,25]. The gene expression pattern of largemouth bass was significantly changed after long-term exposure to severe hypoxia. Under severe hypoxia, lipolysis was enhanced and anabolism was inhibited, leading to a significant decrease in hepatic fat droplet area, and the cell cycle was arrested in the G1 phase with DNA replication suspended [21]. The studies of these adaptive mechanisms provided an important basis for understanding the survival strategies of largemouth bass in different environments. Although some of the adaptive mechanisms have been revealed, the adaptive differences and molecular basis of largemouth bass still need to be further explored, which will help to improve the economic benefits of local aquaculture.

In this study, largemouth bass in different environments were collected as the materials, one group was from Huzhou City (120.09°E, 30.7°N), Zhejiang Province, China, and the other was introduced from Huzhou to Xingtai City (114.68°E, 37.01°N), Hebei Province, China. Huzhou and Xingtai are geographically located in the subtropical monsoon climate and temperate monsoon climate zones, respectively. There are distinct differences in climatic conditions, such as temperature, humidity and sunlight duration. Largemouth bass can survive normally in Xingtai, but the condition factor showed a declining trend. To analyze the adaptive mechanism of largemouth bass at the transcriptional level, the gene expression differences of largemouth bass adapted to local breeding environments were analyzed by comparative transcriptomics.

## 2. Materials and Methods

### 2.1. Sample Collection

A total of twelve largemouth bass samples were collected from Xingtai and Huzhou, with an equal distribution of six samples from each location, comprising three females and three males. All samples were bred in local outdoor ponds at a density of about 60,000 per hectare and fed with compound feed produced by Zhejiang Huzhou Yisheng Feed Company (Huzhou, China). The average temperature in Huzhou ranged from 15 °C to 23 °C, while in Xingtai, it ranged from 9 °C to 21 °C. Compared to Huzhou, Xingtai had relatively lower temperatures, especially during the night. The sunshine duration in Xingtai was shorter than that in Huzhou, which also led to differences in water temperature. Four groups were set up: female group in Huzhou (HZ-F: HZ-F1, HZ-F2, HZ-F3), male group in Huzhou (HZ-M: HZ-M1, HZ-M2, HZ-M3), female group in Xingtai (XT-F: XT-F1, XT-F2, XT-F3) and male group in Xingtai (XT-M: XT-M1, XT-M2, XT-M3). Two comparison groups were set up, one was a female group (HZ-F vs. XT-F) and the other was a male group (HZ-M vs. XT-M). In each comparison group, the former was a control group and the latter was an experimental group.

### 2.2. cDNA Library Preparation and Sequencing

The samples were in the mature stage, with an average weight of 554 g and an average body length of 29.5 cm. The back muscle tissues were used to prepare RNA. Total RNA was extracted according to the instruction manual of the TRlzol reagent (Life technologies, Carlsbad, CA, USA). RNA concentration and purity were measured using a NanoDrop 2000. RNA integrity was evaluated using the RNA Nano 6000 Assay Kit of the Agilent Bioanalyzer 2100 system (Agilent, Santa Clara, CA, USA). Each RNA sample preparation was initiated with a total of 1 μg RNA per sample as the input material. Sequencing libraries were generated using a Hieff NGS Ultima Dual-mode mRNA Library Prep Kit for Illumina, and index codes were added to attribute sequences to each sample. The libraries were sequenced on the Illumina NovaSeq platform, yielding 150 bp paired-end reads. The raw reads were further processed using a bioinformatics pipeline tool to remove reads containing adapters, poly-N sequences and low-quality reads. After data processing, the raw sequences were converted into clean reads. All subsequent analyses were conducted using the high-quality clean reads.

### 2.3. Transcriptome Analysis

The clean reads were aligned to the reference genome sequence, and only those reads with a perfect match were selected for further analysis. These reads were then annotated based on the reference genome. Hisat2 [26] (Version 2.1.0) was used to quickly and accurately map the reads with the reference genome to obtain the location information of reads on the reference genome. StringTie [27] (Version 2.2.1) was used to assemble the above reads, and the transcriptome was reconstructed for subsequent analysis. Gene function was annotated based on NCBI non-redundant protein sequences (Nr), protein family (Pfam), Clusters of Orthologous Groups of proteins (KOG/COG), Swiss-Prot (a manually annotated and reviewed protein sequence database), KEGG Ortholog (KO) and Gene Ontology (GO) databases.

### 2.4. Analysis of Gene Expression Levels and Alternative Splicing Genes

StringTie [27] was used to implement the maximum traffic algorithm and fragments per kilobase of transcript per million fragments mapped (FPKM) were used for standardization. The FPKM method effectively mitigated the impact of gene length and sequencing quantity on gene expression calculations. This allowed for the direct comparison of gene expression values. For the differential alternative splicing (AS) analysis, the ASprofile tool [28] was employed to identify and quantify variations in splicing patterns among samples.

### 2.5. Analysis of Gene Expression Differences

Genes with significantly different expression levels in different samples are called differentially expressed genes (DEGs). DESeq2 [29] (Version 1.24.0) was used to perform differential expression analysis between groups. DESeq2 fit a negative binomial distribution model to normalized transcriptomic data, estimated the variability of gene expression and identified genes with significantly changed expression under different conditions using likelihood ratio tests. For the significantly differentially expressed genes, fold change ≥ 2 and FDR < 0.05 were used as screening criteria. Fold change represented the ratio of expression between two groups. False discovery rate (FDR) was obtained by correcting for the difference significance *p*-value, indicating the significance of the difference.

### 2.6. Functional Analysis of Differentially Expressed Genes

Functional enrichment analysis of DEGs was conducted using the Gene Ontology (GO) and Kyoto Encyclopedia of Genes and Genomes (KEGG) databases. The GO system is structured around three principal categories: biological processes, molecular functions and cellular components, with each category represented by specific terms that correspond to particular attributes. Additionally, significant enrichment within KEGG pathways was employed to pinpoint the key biochemical and metabolic pathways, as well as signal transduction pathways.

## 3. Results

### 3.1. Statistics of High-Throughput Sequencing Data

Following the sequencing quality control process, a total of 86.85 Gb of clean data was acquired for the transcriptome analysis of 12 largemouth bass samples, with each sample exhibiting a Q30 of no less than 94.85% (Table 1).

### 3.2. Alternative Splicing (AS) Analysis

**Figure 1:**
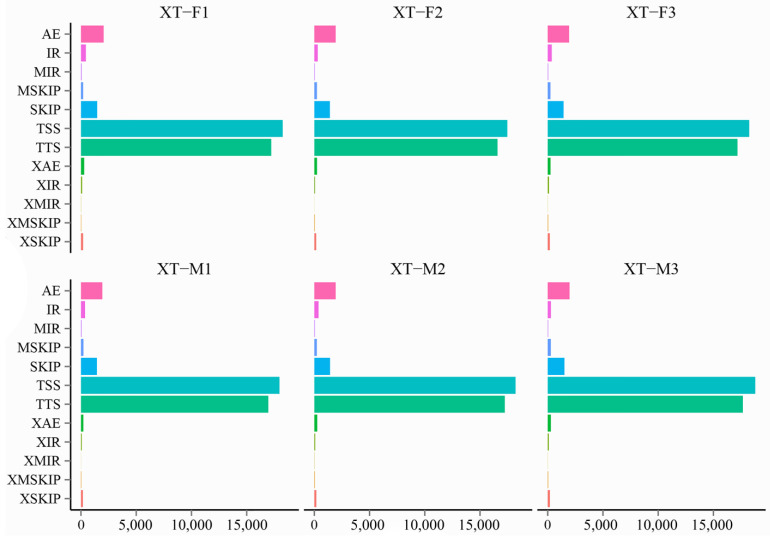
AS events of largemouth bass in Xingtai. The horizontal axis represents the number of variable clippings, and the vertical axis represents the classification of variable clippings. TSS: alternative 5′ first exon (transcription start site); TTS: alternative 3′ last exon (transcription terminal site); SKIP: skipped exon (SKIP_ON, SKIP_OFF pair); XSKIP: approximate SKIP (XSKIP_ON, XSKIP_OFF pair); MSKIP: multiexon SKIP (MSKIP_ON, MSKIP_OFF pair); XMSKIP: approximate MSKIP (XMSKIP_ON, XMSKIP_OFF pair); IR: intron retention (IR_ON, IR_OFF pair); XIR: approximate IR (XIR_ON, XIR_OFF pair); MIR: multi-IR (MIR_ON, MIR_OFF pair); XMIR: approximate MIR (XMIR_ON, XMIR_OFF pair); AE: alternative exon ends (5′, 3′ or both); XAE: approximate AE.

Among the 26,719 genes in the largemouth bass, a total of 39,065 AS events were detected in these genes from six samples from Xingtai. The most prevalent AS event was alternative 5′ first exon (TSS) at 45.47% of all events, followed by alternative 3′ last exon (TTS) at 42.93%, alternative exon ends (AEs) at 4.91%, skipped exons (SKIP) at 3.63%, intron retention (IR) at 0.90%, approximate AE (XAE) at 0.65%, multiexon SKIP (MSKIP) at 0.59%, approximate SKIP (XSKIP) at 0.46%, approximate IR (XIR) at 0.23%, multi-IR (MIR) at 0.13%, approximate MSKIP (XMSKIP) at 0.09% and approximate MIR (XMIR) at 0.03%. The distribution of these AS events in genes of the Xingtai group is depicted in Figure 1 and Table S1.

### 3.3. Differentially Expressed Gene (DEG) Analysis

**Figure 2:**
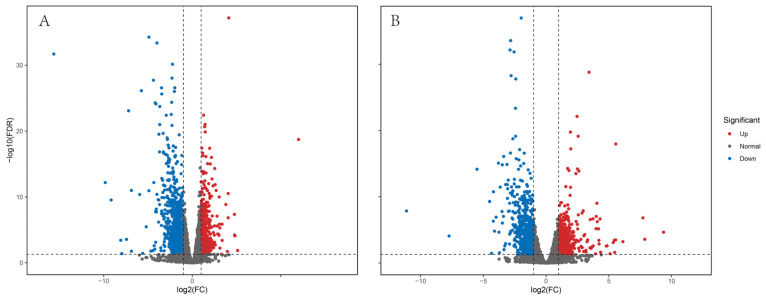
Differentially expressed genes in groups of females ((**A**): HZ-F vs. XT-F) and males ((**B**): HZ-M vs. XT-M).

With fold change ≥ 2 and FDR < 0.05 as the criteria for differential gene screening, differentially expressed genes of each group (HZ-F vs. XT-F, HZ-M vs. XT-M) were screened. Largemouth bass from Huzhou were taken as the control group. The number of DEGs is statistically shown in Table 2, Figure 2. The number of DEGs between Huzhou and Xingtai female groups (HZ-F vs. XT-F) was 1678, including 541 upregulated genes and 1137 downregulated genes, and the number of downregulated genes was about twice the number of upregulated genes. The number of DEGs between Huzhou and Xingtai male groups (HZ-M vs. XT-M) was 1287, including 542 upregulated genes and 745 downregulated genes.

**Figure 3:**
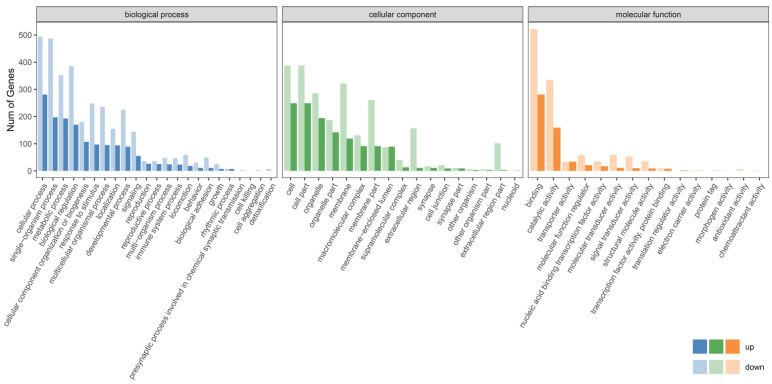
GO function enrichment of DEGs in the female group.

**Figure 4:**
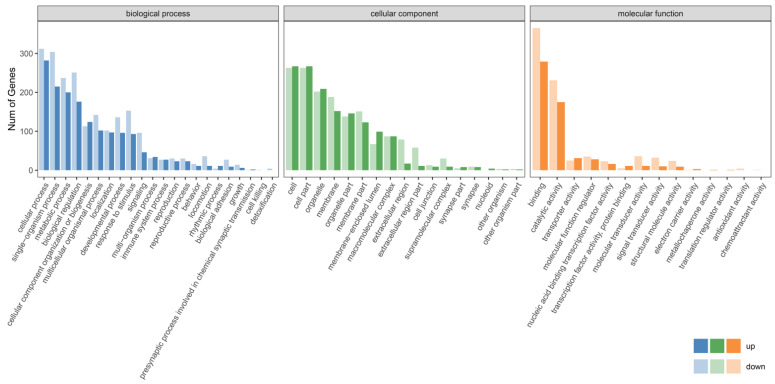
GO function enrichment of DEGs in the male group.

GO enrichment analysis showed that DEGs in largemouth bass were distributed in the three modules of biological process, cell component and molecular function, and the number of genes in downregulated GO terms was higher (Figure 3 and Figure 4, Tables S2–S5). In the biological process term, the DEGs of the female group were mainly enriched in the cellular process, single–organism process, metabolic process, biological regulation, cellular component organization or biogenesis, response to stimulus, multicellular organismal process, localization, developmental process, signaling, reproduction and immune system process (Figure 3, Tables S2 and S3). In the cellular component term, DEGs were mainly enriched in the cell, organelle, membrane, macromolecular complex and supramolecular complex, extracellular region, synapse and cell junction (Figure 3, Tables S2 and S3). In the molecular function term, DEGs were mainly enriched in binding, catalytic activity, transporter activity, molecular function regulator, nucleic acid binding transcription factor activity, molecular transducer activity, signal transducer activity, structural molecule activity, transcription factor activity, translation regulator activity, electron carrier activity and antioxidant activity (Figure 3, Tables S2 and S3).

In the biological process term, the DEGs of the male group were mainly enriched in the cellular process, single–organism process, metabolic process, biological regulation, cellular component organization or biogenesis, multicellular organismal process, localization, developmental process, response to stimulus, signaling, immune system process, reproduction, behavior, locomotion, growth and detoxification (Figure 4, Tables S4 and S5). In the cellular component term, DEGs were mainly enriched in the cell, organelle, membrane, macromolecular complex, extracellular region, cell junction, supramolecular complex and synapse (Figure 4, Tables S4 and S5). In the molecular function term, DEGs were mainly enriched in binding, catalytic activity, transporter activity, molecular function regulator, nucleic acid binding transcription factor activity, transcription factor activity, molecular transducer activity, signal transducer activity, structural molecule activity, electron carrier activity, metallochaperone activity, translation regulator activity and antioxidant activity (Figure 4, Tables S4 and S5).

**Figure 5:**
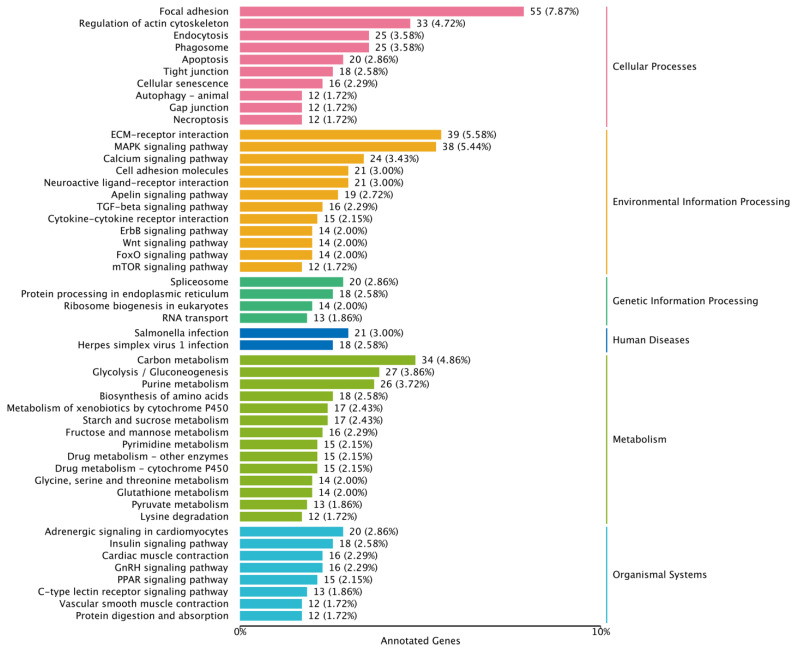
KEGG function enrichment of DEGs in the female group.

KEGG enrichment analysis showed that DEGs were mainly enriched in metabolism, cellular processes, environmental information processing and organismal systems. The DEGs of the female group were mainly enriched in carbon metabolism, glycolysis/gluconeogenesis, purine metabolism, biosynthesis of amino acids, metabolism of xenobiotics by cytochrome P450, starch and sucrose metabolism, fructose and mannose metabolism, pyrimidine metabolism, focal adhesion, regulation of actin cytoskeleton, endocytosis, phagosome, ECM–receptor interaction, MAPK signaling pathway, adrenergic signaling in cardiomyocytes, insulin signaling pathway, cardiac muscle contraction, PPAR signaling pathway, spliceosome, protein processing in endoplasmic reticulum and ribosome biogenesis in eukaryotes (Figure 5).

**Figure 6:**
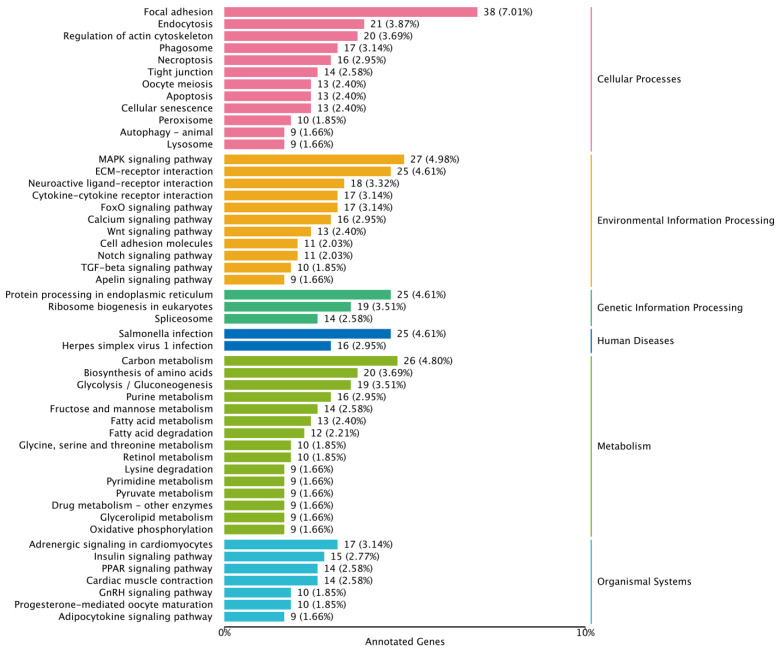
KEGG function enrichment of DEGs in the male group.

The DEGs of the male group were mainly enriched in carbon metabolism, biosynthesis of amino acids, glycolysis/gluconeogenesis, purine metabolism, fructose and mannose metabolism, focal adhesion, endocytosis, regulation of actin cytoskeleton, MAPK signaling pathway, ECM–receptor interaction, adrenergic signaling in cardiomyocytes, insulin signaling pathway, PPAR signaling pathway, cardiac muscle contraction, protein processing in endoplasmic reticulum and ribosome biogenesis in eukaryotes (Figure 6).

## 4. Discussion

The largemouth bass holds significant economic value, and research on its adaptability mechanisms has consistently been a hot topic [30]. Temperature, water quality and oxygen levels are all important factors that affect the fitness of aquaculture [13,14,15,17]. In our previous study, we observed that, after transferring largemouth bass from Huzhou to Xingtai and breeding them in the laboratory for 115 days, the weight and length increased significantly, but their condition factor showed a declining trend. However, the largemouth bass from Huzhou continued to grow well. That was related to the different breeding environment.

Environmental changes triggered stress responses, which in turn impacted the breeding condition factor [31,32,33]. To explore the adaptability of largemouth bass to environmental changes, we analyzed the alternative splicing genes, differentially expressed genes and their functions. The transcriptome data revealed that there were numerous alternatively spliced genes in the largemouth bass from Xingtai (Figure 1), which might play a key role in environmental adaptation [34,35]. The comparative transcriptomic analysis results showed that the largemouth bass from Xingtai presented a large number of DEGs in both female and male groups (Table 2). In the female group, 1678 DEGs were detected, with 541 upregulated and 1137 downregulated, indicating that the number of downregulated genes was approximately twice as high as that of upregulated genes. In the male group, 1287 DEGs were detected, with 542 upregulated and 745 downregulated. The DEGs were mainly enriched in biological processes, such as the metabolic process, biological regulation, response to stimulus, developmental process, signaling, reproduction and immune system process (Figure 3 and Figure 4). These DEGs were involved in pathways including carbon metabolism, glycolysis/gluconeogenesis, purine metabolism, biosynthesis of amino acids, starch and sucrose metabolism, fructose and mannose metabolism, pyrimidine metabolism, MAPK signaling pathway, spliceosome, protein processing in the endoplasmic reticulum and ribosome biogenesis in eukaryotes (Figure 5 and Figure 6). According to previous research, genes associated with growth were notably enriched in pathways involved in lipid metabolism, whereas those responsive to temperature changes were predominantly connected to carbohydrate metabolism [36]. In our study, signaling pathways related to fatty acid metabolism were enriched in the male group, but not in the female group, indicating that the utilization pattern of fatty acids might vary by sex. The energy supply pattern of largemouth bass might change, particularly in aspects such as carbon metabolism, glycolysis/gluconeogenesis, starch and sucrose metabolism and fructose and mannose metabolism [37]. Amino acid metabolism played a crucial supporting role in responding to acute hypoxia stress, and our findings revealed an enrichment of several amino acid metabolic pathways, such as purine metabolism, biosynthesis of amino acids, pyrimidine metabolism and glycine, serine and threonine metabolism, which might indicate that largemouth bass suffered from severe hypoxic stress [38]. Interestingly, the glutathione metabolism was enriched in the female group, which was associated with antioxidant processes [39]. The spliceosome pathway was significantly enriched, suggesting that activated alternative splicing was a key biological process that helped fish adapt to environmental changes [40,41,42]. Under heat stress, the weight gain rate and specific growth rate of largemouth bass decreased, the liver tissue was damaged and the chronic heat stress was coped with by enhancing the glycolytic pathway, inhibiting the gluconeogenesis pathway and lipid metabolism [2,43]. Under low-temperature conditions, largemouth bass undergoing out-of-season reproduction altered sex hormone synthesis, angiogenesis and lipid deposition, thereby delaying ovarian development [14]. In general, the results of functional enrichment of DEGs revealed that most signaling pathways were related to metabolism, such as carbon metabolism, biosynthesis of amino acids, glycolysis/gluconeogenesis and fatty acid metabolism, indicating that temperature was one of the key factors affecting the growth performance differences between largemouth bass in Xingtai and Huzhou [36]. The KEGG functional enrichment analysis revealed that the focal adhesion (FA) signaling pathway contained the highest number of DEGs. The survival of endothelial cells relied on the interactions between the extracellular matrix and integrins, which were mediated through focal adhesions [44,45]. Most focal adhesion molecules were mainly expressed on the gill, skin and intestinal immune organs, which showed important effects on antimicrobial immunity, adhesion, signaling, force transduction and regulation of the cytoskeleton [46,47]. Importantly, in our study, the pathways of regulation of the cytoskeleton, endocytosis, phagosome and tight junction were also enriched, suggesting that the adaptability of largemouth bass has been affected. More signal pathways related to environmental information processing were enriched, such as the MAPK signaling pathway, ECM–receptor interaction and calcium signaling pathway [48,49,50]. According to the previous studies, immune-related dimers and genes related to metabolic processes in the FA and ECM–receptor interaction (ECM-RI) pathways were downregulated after infection with largemouth bass ricovirus (MSRV), which might be related to gut microbiota [51]. And the upregulation of ECM-RI and other immune-related DEGs contributed to resisting infection with *A. hydrophila* [52]. In addition, the stress of metal ions can also affect the expression of the FA pathway. Under cupric ion stress, the FA pathway was significantly upregulated in crayfish (*Procambarus clarkii*), and hub genes in the pathway played a key role in crayfish’s response to the stress of metal ions [53]. In our study, the functional enrichment results of DEGs indicated that largemouth bass might adjust energy supply, immune responses and stress reactions by regulating pathways such as focal adhesion, carbon metabolism, sugar metabolism, amino acid metabolism and the spliceosome, thereby adapting to the environment. In future research, we will further focus on disease resistance, gut microbiota and population evolution under different environmental conditions, which are crucial areas for understanding adaptability and health.

## 5. Conclusions

The largemouth bass has always been a focus of research regarding its adaptability, which is closely related to the economic benefits of aquaculture. To explore the adaptability of largemouth bass to different environments, this study comparatively analyzed the transcriptome data of largemouth bass from Xingtai and their native place, Huzhou. The results showed that the DEGs were involved in processes such as growth, metabolism, development, immunity and stress response. We speculated that largemouth bass might adjust the process of energy supply, immune responses and stress reactions by regulating the expression of genes related to metabolism, stress and immunity to maintain physiological function and adapt to the environment. Our research provided evidence for the study of largemouth bass adaptability at the molecular level. Next, we will continue to focus on the specific factors affecting the adaptability of largemouth bass.

## Figures and Tables

**Table 1:** Transcriptome sequencing data of largemouth bass.

Group	Sample	Total Reads	ReadSum	BaseSum	GC (%)	Q30 (%)
Female group in Huzhou (HZ-F)	HZ-F1	52,980,370	26,490,185	7,930,807,418	50.90	95.29
	HZ-F2	52,141,432	26,070,716	7,799,815,066	50.84	95.43
	HZ-F3	42,252,350	21,126,175	6,324,348,192	51.19	95.16
Male group in Huzhou (HZ-M)	HZ-M1	46,796,218	23,398,109	7,004,969,356	50.95	94.85
	HZ-M2	44,605,728	22,302,864	6,677,397,946	51.19	95.15
	HZ-M3	49,471,502	24,735,751	7,406,998,266	51.00	94.88
Female group in Xingtai (XT-F)	XT-F1	53,488,148	26,744,074	8,001,807,860	50.50	95.37
	XT-F2	48,387,164	24,193,582	7,243,791,296	50.72	95.08
	XT-F3	48,131,874	24,065,937	7,199,491,898	50.62	95.23
Male group in Xingtai (XT-M)	XT-M1	51,856,652	25,928,326	7,759,384,482	50.71	95.48
	XT-M2	40,941,278	20,470,639	6,128,805,602	50.35	94.91
	XT-M3	49,290,250	24,645,125	7,374,428,542	50.32	95.60

**Table 2:** The number of DEGs between Huzhou and Xingtai largemouth bass.

DEG Set	DEG Number	Upregulated	Downregulated
HZ-F vs. XT-F	1678	541	1137
HZ-M vs. XT-M	1287	542	745

## Data Availability

Data will be made available on request. The raw RNA-seq data (Accession no. PRJEB86064) were uploaded to ENA.

## Supplementary Materials

The following supporting information can be downloaded at: https://www.mdpi.com/article/10.3390/genes16030267/s1, Table S1: Statistics of largemouth bass AS events in Xingtai; Table S2: Upregulated DEGs GO enrichment in female group; Table S3: Downregulated DEGs GO enrichment in female group; Table S4: Upregulated DEGs GO enrichment in male group; Table S5: Downregulated DEGs GO enrichment in male group.
